# Impact of surgical parathyroidectomy on chronic kidney disease-mineral and bone disorder (CKD-MBD) – A systematic review and meta-analysis

**DOI:** 10.1371/journal.pone.0187025

**Published:** 2017-11-06

**Authors:** Mugurel Apetrii, David Goldsmith, Ionut Nistor, Dimitrie Siriopol, Luminita Voroneanu, Dragos Scripcariu, Marc Vervloet, Adrian Covic

**Affiliations:** 1 University of Medicine and Pharmacy "Grigore T. Popa", Iasi, Romania; 2 Department of Nephrology “Dr CI Parhon” Hospital, Iasi, Romania; 3 Renal, Dialysis and Transplantation Unit, Guy’s and St Thomas’ Hospital, London, United Kingdom; 4 ERBP Methods Support Team, Ghent University Hospital, Ghent, Belgium; 5 Surgery Department, Regional Institute of Oncology, Iasi, Romania; 6 Department of Nephrology and Amsterdam Cardiovascular Sciences, VU University Medical Center, Amsterdam, Netherlands; The University of Tokyo, JAPAN

## Abstract

For more than 6 decades, many patients with advanced chronic kidney disease (CKD) have undergone surgical parathyroidectomy (sPTX) for severe secondary hyperparathyroidism (SHPT) mainly based historical clinical practice patterns, but not on evidence of outcome.We aimed in this meta-analysis to evaluate the benefits and harms of sPTX in patients with SHPT. We searched MEDLINE (inception to October 2016), EMBASE and Cochrane Library (through Issue 10 of 12, October 2016) and website clinicaltrials.gov (October 2016) without language restriction. Eligible studies evaluated patients reduced glomerular filtration rate (GFR), below 60 mL/min/1.73 m^2^ (CKD 3–5 stages) with hyperparathyroidism who underwent sPTX. Reviewers working independently and in duplicate extracted data and assessed the risk of bias. The final analysis included 15 cohort studies, comprising 24,048 participants. Compared with standard treatment, sPTX significantly decreased all-cause mortality (RR 0.74 [95% CI, 0.66 to 0.83]) in End Stage Kidney Disease (ESKD) patients with biochemical and / or clinical evidence of SHPT. sPTX was also associated with decreased cardiovascular mortality (RR 0.59 [95% CI, 0.46 to 0.76]) in 6 observational studies that included almost 10,000 patients. The available evidence, mostly observational, is at moderate risk of bias, and limited by indirect comparisons and inconsistency in reporting for some outcomes (eg. short term adverse events, including documented voice change or episodes of severe hypocalcaemia needing admission or long-term adverse events, including undetectable PTH levels, risk of fractures etc.). Taken together, the results of this meta-analysis would suggest a clinically significant beneficial effect of sPTX on all-cause and cardiovascular mortality in CKD patients with SHPT. However, given the observational nature of the included studies, the case for a properly conducted, independent randomised controlled trial comparing surgery with medical therapy and featuring many different outcomes from mortality to quality of life (QoL) is now very strong.

## Introduction

For many decades surgical parathyroidectomy (sPTX) has been a well-recognised potential clinical intervention for patients with chronic kidney disease (CKD) with unremitting secondary hyperparathyroidism (SHPT), especially for those patients receiving long-term renal replacement therapy [1, 2]. Approximately 5–10 percent of patients with end-stage kidney disease (ESKD) undergo sPTX for severe SHPT [3]; this percentage significantly increases for long-term dialysis survivors. Typically, such patients have biochemical and radiological abnormalities related to SHPT, and important extra skeletal pathology, mostly cardiovascular [4]. Over the last two decades, with the widespread and targeted use of different types of vitamin D therapy, and / or oral calcimimetics, which represent the “medical management” of SHPT, the sPTX rate was expected to fall substantially[5] [6], Two recent reports present contradictory findings in this respect. The first one coming from the DOPPS cohort shows a general trend for increased serum PTH concentrations over the time period covered, while the sPTX rates declined in all regions (1996–2011) [7]. The second study derived from the Healthcare Cost and Utilization Project’s Nationwide Inpatient Sample—a representative national database on hospital stay, coupled with parathyroidectomy data from the US Renal Data System (2002–2011) shows a fairly static rate of sPTX in the US between 2002 and 2011[8].

Currently, it is not yet clear if there is a morbidity-mortality benefit following sPTX for severe SHPT in ESKD. There is a clear potential anaesthetic-surgical risk involved and the DOPPS data referred to above also showed an increased mortality hazard ratio with ***low*** serum PTH concentrations (the natural outome of successful parathyroid gland surgery). At the same time, recent guideline recommendations by latest European Renal Best Practice (ERBP) statement downgraded the use of calcimimetics [9]; thus the optimal therapeutic strategy for patients with biochemical changes but without clinical symptoms of SHPT, remains unclear. Whether or not to perform sPTX in CKD and ESKD patients remain a current, controversial and challenging issue. Therefore, clinicians may benefit from a critical systematic summary of the best available evidence regarding the benefits and risks associated with this intervention, to advise their patients accordingly. We conducted a meta-analysis of available evidence to assess the impact of sPTX on the outcomes of CKD /ESKD patients with SHPT compared with matched patients not undergoing sPTX.

## Methods

The systematic review and meta-analysis was performed according to a previously published protocol (registration number:CRD42017067736). https://www.crd.york.ac.uk/PROSPERO/display_record.asp? ID = CRD42017067736

### Purpose

This review aims to evaluate the benefits and harms of performing PTX in CKD/ESKD patients with secondary hyperparathyroidism.

### Data sources/search strategy

We searched MEDLINE (inception to October 2016), the Cochrane Library (Issue 10–12, October 2016) and the website clinicaltrials.gov (October 2016) and EMBASE without language restriction. Hand search for relevant articles was done on reference lists from textbooks, articles, and scientific proceedings. The search terms used and a detailed search strategy is available in the S1 Table.

### Study selection

We conducted a systematic review and meta-analysis on observational cohorts studies and randomized controlled trials (RCTs) in adults with CKD 3–5 stages (GFR below 60 mL/min/1.73 m^2^)) that evaluated the role of parathyroidectomy in determining clinical outcomes in patients with CKD-MBD. Studies enrolling any patient with CKD stages 3–5 (as defined by the Kidney-Disease Outcomes and Quality Initiative [K-DOQI] guidelines: stage 3 = GFR 30–59 ml/min/1.73 m2; stage 4 = GFR 15–29 ml/min/1.73 m2; stage 5 = GFR <15 ml/min/1.73 m2 including those requiring dialysis) with evidence of secondary hyperparathyroidism who had undergone parathyroid surgery were included in this analysis. The surgery itself could be (1) total parathyroidectomy without auto transplantation, (2) total parathyroidectomy with auto transplantation, or, (3) subtotal parathyroidectomy. Patients were compared with control CKD/ESKD patients with non-surgical treatment for SHPT. Patients with CKD/ESKD undergoing surgery for primary hyperparathyroidism and also those undergoing re-operative parathyroidectomy, were excluded.

### Data extraction and synthesis

Data extraction was done independently by two authors (IN and MA) using standard data extraction forms. When more than one publication of one study was found, reports were grouped together and only the publication with the most complete data was included. Data extracted included identifying information, aim of the study, details of the study protocol and demographic data. We extracted characteristics of each study including baseline PTH values, baseline clinical characteristics of the study population, known comorbidities, type of study design, types of surgery and use of agents interfering with PTH release and total duration of follow-up. Any unclear or missing information was requested from the authors by written correspondence and any relevant information obtained was included in the review. Disagreements were resolved by consultation between all authors.

### Risk of bias

Two reviewers (MA and IN) evaluated the quality of the selected studies independently without blinding to authorship or journal according to recommendations from the Cochrane Collaboration. The quality items assessed were selection bias (random sequence generation, allocation concealment), performance bias (blinding of patients and investigators), detection bias (blinding of outcome assessors), attrition bias (incomplete outcome data), reporting bias (selective reporting) and other forms of bias (significant different group comparisons, funding sources, early termination of a trial). For the observationa studies, the quality was assessed using the Newcastle-Ottawa scale (NOS)[10]. The scale used three categories to evaluate: selection of the study groups, the comparability of the groups and the assesement of outcome. Stars awarded for each quality item serve as a quick visual assessment. Stars are awarded such that the highest quality studies are awarded up to nine stars. Disagreements were resolved by consensus. Publication bias was assessed using the funnel plot technique [11].

### Main outcomes and measures

Primary outcomes of this analysis were all-cause mortality: short term and long-term and cardio-vascular mortality from the time of the surgical intervention to the end of follow-up. Secondary outcomes were: (1) QoL, (2) short term adverse events, including documented voice change or episodes of severe hypocalcaemia needing admission, (3) long-term adverse events, including “aparathyroid state” (undetectable PTH levels), fractures, and, (4) postoperative PTH levels. These are all outcomes of definite clinical relevance and importance. Summarized treatment effects were analysed for mortality and cardiovascular (CV) mortality, both postoperative and long-term, using random-effects meta-analysis.

### Statistical analysis

We summarized effect estimates using standard and cumulative random effects meta-analysis. We used a random-effects model for meta-analysis and expressed treatment effects as a risk ratio (RR) with 95% confidence intervals (CI).[12]. We used the *I*^*2*^ statistic to assess for inconsistency across individual studies[12]. An *I*^*2*^>50% indicated large inconsistency across studies (heterogeneity) not explained by chance[13]. We considered a p-value below 0.10 to indicate significant heterogeneity. In cumulative meta-analysis, outcome data for all-cause mortality and cardiovascular mortality from all available trials were included sequentially according to the year in which they first became available. All analyses were performed using Review Manager Version 5.2 (The Cochrane Collaboration 2012) and Stata SE software, version 12 (StataCorp. Stata Statistical Software: Release 12. College Station, TX: StataCorp LP.) [14].

Additional prespecified subgroup analyses were conducted to explore potential causes of heterogeneity for treatment effect on all-cause mortality. Treatment heterogeneity was analysed also in relation with prior medical treatment. The following factors were planned to be investigated in subgroup analyses (1) publication date, (2) use of calcimimetics in the perioperative period, and (3) initial PTH levels (<800 pg/ml vs>800 pg/ml). For all analyses, a two-tailed p-value<0.05 indicated statistical significance.

## Results

**Fig 1:**
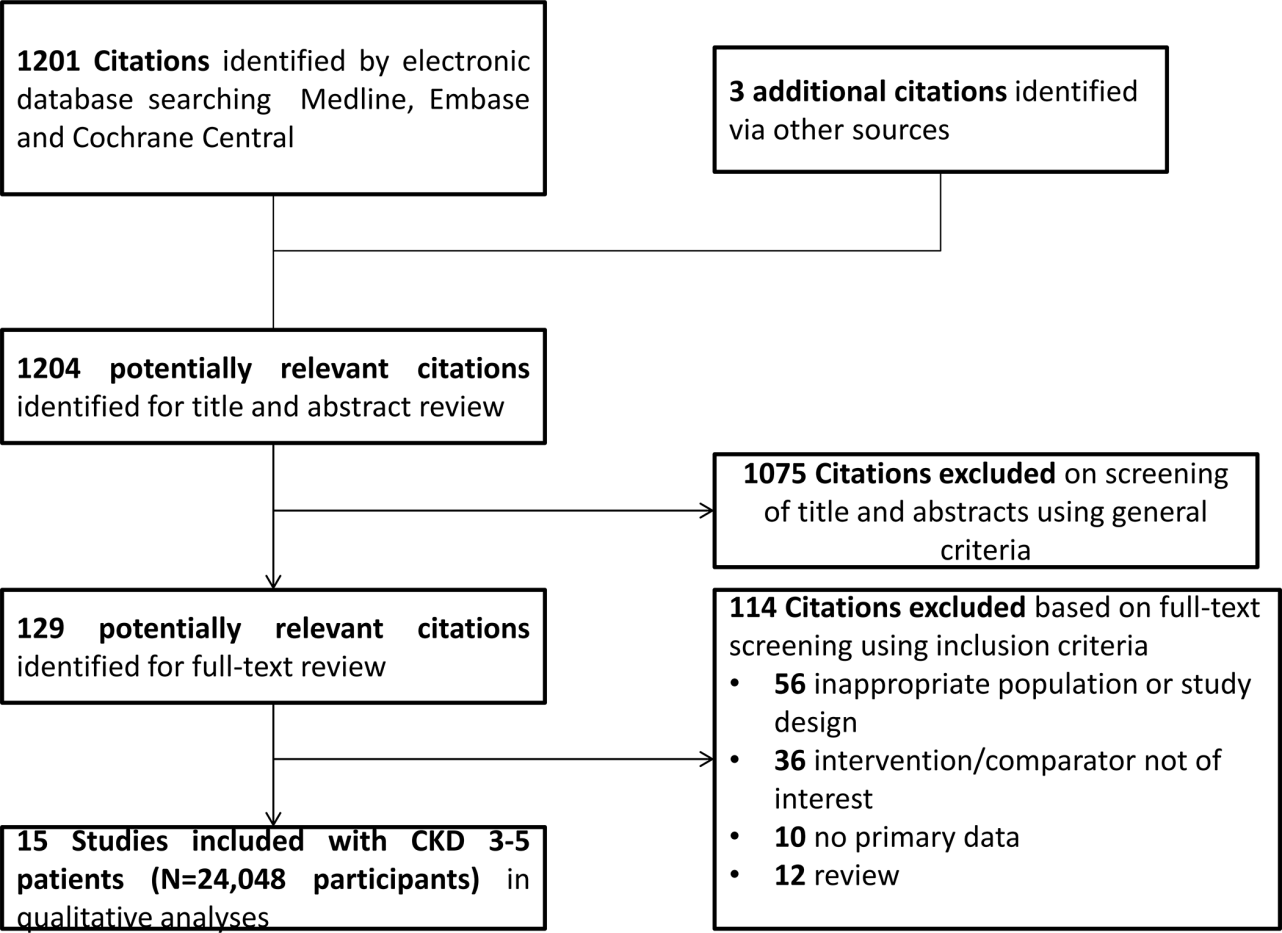
Selection and description of studies.

The literature search identified a total of 997 abstracts, of which 15 observational studies comprising 24,048 participants were selected (Fig 1). No randomized cotrolled trial was indentified. The follow-up period varied between 12 and 360 months. Baseline characteristics of the included studies are listed in Tables 1 and 2. These studies included only prevalent dialyzed patients diagnosed with SHPT who underwent subtotal or total parathyroidectomy with or without auto-transplantation. In the majority of included studies, the parathyroidectomy group was compared to matched controls, who had not undergone parathyroidectomy or refused surgery for various reasons and were conservatively managed. In other studies a propensity score matching was used trying to decrease the risk of selection bias, while in others parathyroidectomy group was compared with control patients identified from different registries, matched for age, sex, race, diabetes as cause of kidney failure, years on dialysis, and dialysis modality. The main outcome in the included studies was all-cause mortality, defined as death by any cause. Six studies also reported cardiovascular mortality. In three of them[15–17], the cardiovascular events included sudden death, heart failure, myocardial infarction, peripheral vascular disease and cerebrovascular accident, while in two[18, 19] the definition included only sudden death, cerebrovascular accident and myocardial infarction. Only one study lacked a precise definition for cardiovascular mortality[19]. Six studies [16–18, 20–22] reported data regarding treatments that might interfere with parathyroid function (phosphate binders or vitamin D compounds). No data regarding contemporaneous or parallel-group calcimimetic treatment were reported in any of the included studies. Overall, the 15 included studied had an averaged NOS score of 6.2 stars (of maximum 9 stars) (Table 1) due to lack of or unclear description of follow-up time and lost to follow-up in the included studies.

**Table 1:** Demographic and characteristics of studies included in the meta-analysis.

Reference (first author)	Country	Parients No		Age		Gender (male%)		Newcastle-Ottawa score		
		PTX	CTRL	PTX	CTRL	PTX	CTRL	Selection	Comparability	Exposure
Ivarsson etal. 2015 [23]	Sweden	423	1234	55.2	56	48.2	50.1	***	**	**
Komaba et al. 2015 [17]	Japan	4428	4428	59.1 ± 11.6	59.3±12.3	55.8	55.7	***	**	***
Conzo et al. 2013 [20]	Italy	30	20	51.5±10.89	55±11.2	26.7	40	***	*	*
Sharma et al. 2013 [51]	US	150	1044	42.1	42.2	46.7	46.7	***	**	**
Goldstein et al 2013 [21]	Brazil	123	128	46	50	46.3	44.5	***	*	**
Iwamoto et al 2012 [16]	Japan	88	88	60.6±8.4	60.5±8.4	53.4	53.4	***	**	**
Kestenbaun et al. 2004 [24]	US	4558	4558	47.6	47.6	42.5	42.5	***	**	*
Trombetti et al. 2007 [44]	Switzerland	40	80	42.6	55	45	51	***	**	**
Ho LC et al. 2016 [45]	Taiwan	998	998	54.7	55	42.9	42.5	***	**	***
Moldovan et al. 2015 [22]	Romania	26	26	51.62±9.92	49.65±11.49	53.84	23.07	***	*	**
Li-Wedong et al 2016 [46]	China	53	92	63.1±13.8	53.8±15	56.6	70.6	***	*	*
Costa-Hong et al 2007 [18]	Brazil	50	68	52	59	43±10	45±12	**	**	*
Dussol B et al 2007[47]	France	19	32	N/A	N/A	N/A	N/A	**	**	*
Ma T-L et al 2015[48]	Taiwan	60	161	N/A	N/A	N/A	N/A	**	**	*
Lin H-C 2014[19]	Taiwan	30	23	53.3 ± 13.3	53.4 ±13.9	43	61	***	**	*

Abbreviations: PTX-parathyroidectomy, CTRL- control

*- Stars awarded for each quality item (Newcastle-Ottawa scale). For each domain, either a "star" or "no star" is assigned, with a "star" indicating that study design element was considered adequate and less likely to introduce bias. For Selection (of the exposed cohort, of the non-exposed cohort, ascertainment of exposure and outcome of interest) a maximum of four stars may be assigned. A maximum of two stars can be given for Comparability and a maximum of 3 stars can be given for Exposure (assessment of outcome, length of follow-up and adequacy offollow-up). A study could receive a maximum of nine stars.

**Table 2:** Baseline characteristics of the studies included in the meta-analysis.

Reference (first author)	Design of study	Duration of follow-up (months)	Baseline PTH		Type of surgery	Type of control group	Inclusion criteria	Exclusion criteria
			PTX	CTRL				
Ivarsson et al. 2015	Cohort-multicenter- prospective	61.3	N/A	N/A	Total and subtotal PTX	Between one and five patients randomly matched who had not undergone PTX. The matching criteria were birth year in 10-year categories, sex and cause of ESKD in categories (autosomal dominant polycystic kidney disease, diabetes mellitus, glomerulonephritis, nephrosclerosis, pyelonephritis and other/unknown.	Patients on maintenance dialysis and transplantation with SHPT	Errors in reporting of patient information censoring on the same day as initiation of RRT
Komaba et al. 2015	Cohort-multicenter- prospective	12	96 (28–236)	669 (570–870)	Total and subtotal PTX	Propensity score-matched patients who had not despite severe SHPT	≥ 18 years of age with SHPT and were receiving haemodialysis thrice weekly for more than 3 months	No data on demographic characteristics, dialysis prescription, intact PTH levels, or history of PTX
Conzo et al. 2013	One-center retrospective	60	142.08 ± 64.01	102.94 ± 32.51	Total PTX and total PTX with auto-transplantation	Patients with indication for PTX but refusing surgery	SHPT, unresponsive to medical treatment iPTH levels > 53–84, 8 pmol/L, serum P level > 2,09 mmol/l, US enlarged parathyroid glands (> 1 cm or >500 mm3) and persisting clinical symptoms, six months after medical therapy	Renal transplantation,
Sharma et al. 2013	Retrospective and matched-cohort study	33.6	N/A	N/A	Near-total parathyroidectomy	For each NTPTX patient, controls were individually matched for age (±2 years), sex, race, diabetes as cause of end-stage renal disease, dialysis duration (vintage), year they started dialysis (±1 year), and dialysis modality	Prevalent haemodialysis or peritoneal dialysis with SHPT	Kidney transplant, no SHPT, no records on dialysis modality
Goldstein et al 2013	Retrospective cohort study	23	1554	1360	Total parathyroidectomy with auto-transplantation	Patients with refractory SHPT not submitted to PTX	PTH greater than 800 pg/ml on calcitriol or in the presence of hyperphosphatemiaand/or hypercalcemia which prevented the use of calcitriol	Kidney transplant and predialysis patients No SHPT
Iwamoto et al 2012	Retrospective cohort study	53	884.5 ± 388.5	199.0 ± 120.2	Total PTX without autotransplantation	Matched patients for sex, age, underlying disease and prior dialysis history	PTH >500 pg/mL and enlarged parathyroid glands confirmed by imaging, enlarged parathyroid gland with imaging and resistant to reduction of iPTH to below 200 pg/mL for hypercalcemia (corrected Ca>11.0 mg/dL) with VDRAs.	N/A
Kestenbaum et al. 2004	prospective cohort study	53.4	N/A	N/A	Total+subtotal PTX	Individually matched by age, race, gender, cause of ESKD, dialysis duration, prior transplantation status, and dialysis modality	at least 18 years old and had initiated renal replacement therapy with SHPT	Death, lost to follow-up, or underwent PTX during the first 90 days of renal replacement therapy
Trombetti et al. 2007	retrospective cohort study	360	N/A	N/A	Subtotal or total PTX with autotransplantation	two matched controls for each PTX case	ESKD and severe hyperparathyroidism	Kidney transplant, no records, no SHPT
Ho LC et al. 2016	retrospective cohort study	41.52±30.12	N/A	N/A	N/A	The parathyroidectomized patients were matched with the controls based on propensity score for parathyroidectomy	Prevalent dialysis with unremitting SHPT	Renal transplantation prior to dialysis or a history of any kind of malignancy before the initiation of long-term dialysis
Moldovan et al. 2015	prospective cohort study	24	2037	1282	Subtotal or total PTX	patients with iPTH over 700 pg/ml, without surgical intervention and treated with specific drugs	severe sHPT, non-responsive to medical treatment with hypercalcemia and hyperphosphatemia	ESKD patients with SHPT and no parathyroid surgery
Li-Wedong et al 2016	prospective cohort study	12	395.3 ± 332.4	349.8 ± 334.5	N/A	Dialysed patient with SHPT	Age>18 years and less than 70 years old. (Duration of HD is more than 3 months. Patients with SHPT (Based on the 2002 KDOQI)	patients with malignant neoplasms, active tuberculosis, AIDS, receiving kidney transplant surgery within one year, pregnancy or lactation, life expectancy being less than 12 months, acute malnutrition, uncontrolled hypertension, severe anemia, serious liver diseases or interrupted follow-up because of all kinds of reasons
Costa-Hong et al 2007	prospective cohort study	N/A	1278 ±699	1243± 753	Total PTX with autotransplantation in the forearm	Patients who had the diagnosis of medically resistant SHPT and not submitted to PTX	Resistance to medical treatment that was defined as serum levels of parathyroid hormone (PTH) and phosphate greater than 800 pg/mL and 6.5 mg/100 mL, respectively, after a minimum of 6 months of treatment.	Renal transplantation, previous myocardial revascularization, smokers, individuals using lipid-lowering drugs, patients with diabetes, and those with a history of heart failure, stroke, unstable angina, or myocardial infarction within 12 months preceding the initiation of the study
Dussol B et al 2007	prospective cohort study	96	N/A	N/A	Total+subtotal PTX	Patients undergoing chronic hemodialysis treatment	N/A	N/A
Ma T-L et al 2015	Prospective cohort study	36	N/A	N/A	N/A	Hemodialysed patients with iPTH values greater than 800 pg/dL	N/A	N/A
Lin H-C 2014	prospective cohort study	72	1011 ±247	1007 ± 251	total PTX with autograft to the brachioradialis muscle in the forearm without arteriovenous shunt.	ESKD patients who were treated with maintenance haemodialysis and who had intact parathyroid hormone (PTH) levels > 800 pg/ml not receiving PTX	Haemodialysis patients with severe secondary hyperparathyroidism. Severe SHPTH was diagnosed when a patient’s PTH level was higher than 800 pg/ml and was associated with the following symptoms: bone and joint pain, muscle weakness, irritability, itching, bone loss, anaemia resistant to erythropoietin, cardiomyopathy or calciphylaxis.	Switched ti peritoneal dialysis Transfer to other hospital Incomplete medical history Received kidney transplant Not eligible for operation Had previous PTX

Abbreviations: PTH-parathormone, RRT-renal replacement therapy, PTX-parathyroidectomy, SHPT- hyperparathyroidism, ESKD- end–stage kidney disease, VDRAs- vitamin D receptor activators, N/A- not available

### Clinical outcomes

**Fig 2:**
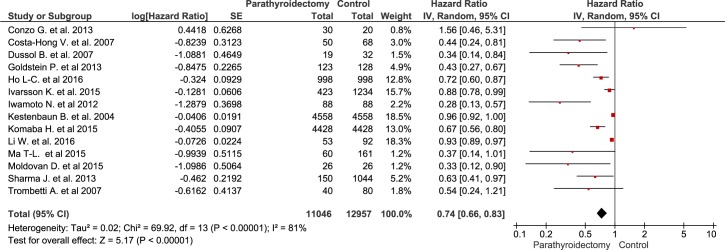
The effect of parathyroidectomy on all-cause mortality.

**Fig 3:**
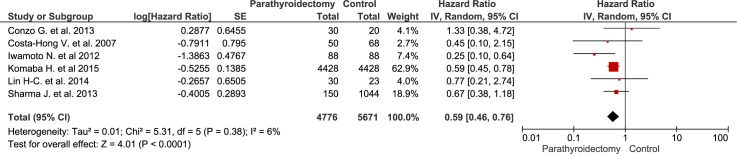
The effect of parathyroidectomy on cardiovascular mortality.

**Fig 4:**
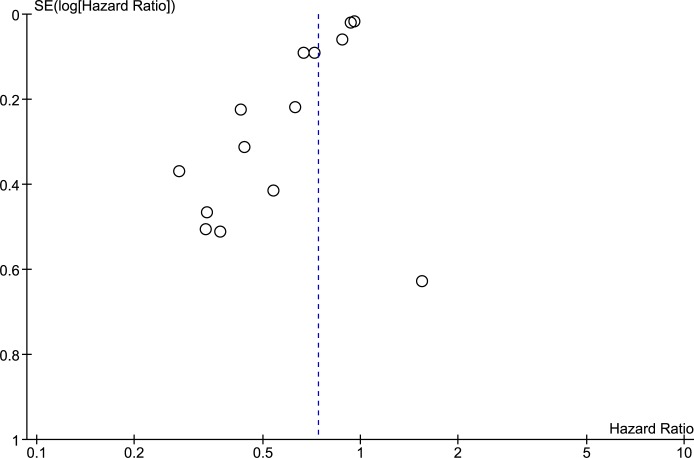
Funnel plot for all-cause mortality.

Compared with standard treatment, sPTX was associated with decreased all-cause mortality (RR 0.74 [95% CI, 0.66 to 0.83) in ESKD patients with secondary hyperparathyroidism (Fig 2). Patients undergoing sPTX had decreased cardiovascular mortality (RR 0.59 [95% CI, 0.46 to 0.76]) in 6 observational studies that included almost 10,000 patients (Fig 3). The heterogeneity across included studies is substantial in the analysis of all-cause mortality (I^2^ = 81%, p < 0.001) and is 6% in the analysis of cardiovascular mortality. The high I^2^ values for all cause mortality show that most of the variability across studies is due to heterogeneity rather than chance. Because the heterogeneity was found to be higher than expected, the model was switched to a random-effect model by calculating the variance of random-effect components. The funnel plot (Fig 4) shows an asymmetrical plot. This was expected in the presence of the important heterogeneity observed for the all-cause mortality outcome, but there is also a suggestion of missing studies in the middle and right of the plot, broadly in the area of non-significance and this could also imply the presence of reporting bias, with smaller negative studies not having been published.

These results were further confirmed by cumulative metanalysis performed in attempt to stratify the studies for trend by time. Cumulative meta-analysis shows that studies conducted since 2012 have collectively signalled benefits for all-cause mortality and since 2015 for cardiovascular mortality (S1 Fig and S2 Fig).

**Fig 5:**
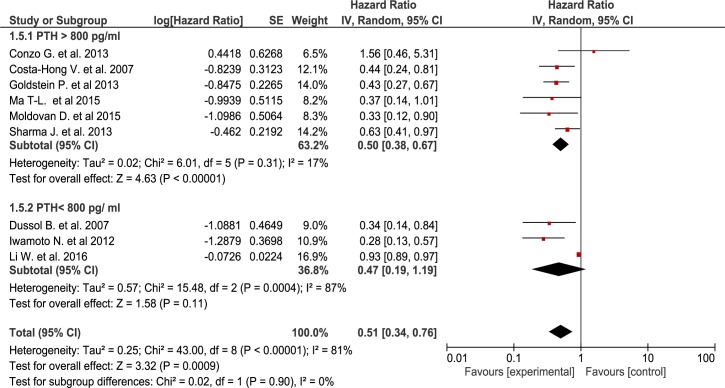
Subgroup analysis for low and high PTH value at baseline.

**Fig 6:**
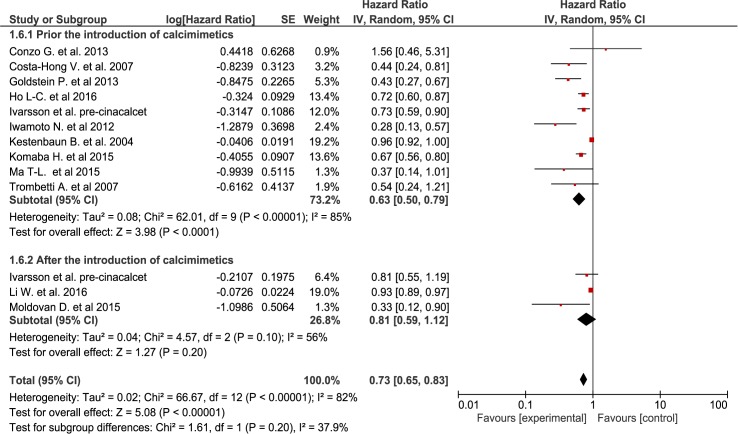
Subgroup analysis according to the moment of calcimimetics introduction.

Giving the significant heterogeneity, subgroup analyses were conducted to explore the effect on mortality for different PTH mean values. Thus, after excluding from the analysis studies with lower PTH (n = 3) or non reported baseline PTH (n = 6), we obtained significantly lower heterogeneicity (Chi^2^ = 6.01, I^2^ = 17%) while maintaining the same significant association with decreased mortality for the sPTX groups (RR = 0.50[95% CI, 0.38 to 0.67))(Fig 5) although the subgroup differences weren’t statiscally significant. Finally, even though no data regarding the actual use of calcimimetics were reported in the included studies, we assessed the impact of cinacalcet approval and introduction on the market. Thus, knowing that the prescription of calcimimetics was increasingly being part of standard medical care of SHPT from the mid 2000s onwards, we performed a subgroup analysis using as a cutoff the date of calcimimetics introduction in clinical practice for different regions. The results were statistically significant only for the pre-calcimimetic era (RR = 0.63 [95% CI, 0.50 to 0.79]), while in the studies performed in the post-calcimimetics era the advantage of sPTX was smaller and lost statistical significance (RR = 0.81 [95% CI, 0.59 to 1.12])(Fig 6). Once again, the test for subgroup differences did not reached statistical significance.

**Fig 7:**
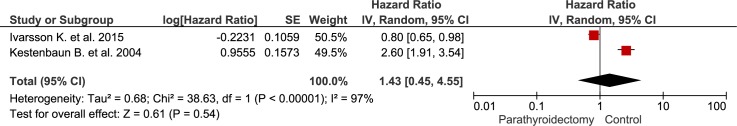
The effect of parathyroidectomy on short-term (30-days) mortality.

Finally we investigated short-term (30 days–peri-operative) mortality which was described in 2 studies only [23, 24]. Although there were more events reported in the parathyroidectomy group, this difference was not statistically significant (RR 1.43 [95% CI, 0.45 to 4.55]) (Fig 7). In one study, the postoperative deaths among PTX patients were mainly cardiovascular (49.3%) and infectious (18.3%), while in the other study, the main causes of short-term mortality were also dominated by myocardial infarction and infections. Regrettably, no data relating to other specific postoperative complications were reported in the included studies.

## Discussion

Evidence derived from 15 observational studies including almost 25,000 patients, suggest that sPTX significantly decreased all-cause mortality in ESKD patients with secondary hyperparathyroidism by almost 30 percent (Fig 2). sPTX had also a positive effect on cardiovascular mortality—a 40 percent reduction in 6 observational studies that included almost 10,000 patients (Fig 3). This positive impact of sPTX compared to standard CKD-MBD management was irrespectively of PTH concentration subgroup at the time of surgery (Fig 5) and was not different in studies conducted after the start of the calcimimetic period in clinical practice.

However, no randomized controlled comparing parathyroid surgery with medical therapy for the treatment of SHPT was found, the final analysis comprising only observational studies with their inherent risk of bias. Heterogeneity was considerable for all-cause mortality and this variation between sample estimates may occur for a variety of reasons, including many study design characteristics, different adjustments for confounding, publication date and real-life populations differences across studies.

sPTX was often regarded by pioneer nephrologists as a “last but necessary resort” option for SHPT but one that would very likely be necessary for many patients surviving on dialysis for more than a few years, without a successful renal transplant. Several observational studies indicate that severe hyperparathyroidism may be associated with increased mortality in this population, presumably via a wide range of cardiovascular, metabolic, hematologic, and immunologic abnormalities induced by high concentrations of the uremic toxin PTH [25–28]. With the advent of an orally-active calcimimetic—cinacalcet—there was an additional medical option, besides active vitamin D and surgical interventions to ameliorate progressive SHPT [29]. Both patients and nephrologists in the “calcimimetic dialysis era” would regard the surgical subtotal extirpation of parathyroid tissue only as an acceptable option for severe, progressive, symptomatic and medically non-responsive secondary hyperparathyroidism.

However, there has never been an RCT comparing medical versus surgically-induced reduction in parathyroid activity in dialysis patients. The publication of the EVOLVE study in 2012 was followed in 2015 by a meta-analysis of the medical benefits and risks of using calcimimetics in SHPT[30]–there was no impact on patient survival or outcomes using cinacalcet in largely asymptomatic, biochemically mild-to-moderate, SHPT[31]. There was no similar meta-analysis assessing the impact of sPTX on hard end-points.

Observational studies, with their obvious risk of bias do not unequivocally prove a clinical benefit from SHPT, but nevertheless may suggest a positive outcome. All reports included in our meta-analysed were cohort studies and national databases/ registries. While acknowledging the important limitations of the current analysis, plausibility of a true beneficial effect on mortality of sPTX comes from theoretical and experimental arguments as well, for instance due to reduction in CV disease following better blood pressure control, and decreased hyperphosphataemia [29, 32] [33]. Other potential benefits of parathyroidectomy include: (1) improvements in mineral bone density and reduced risk of pathological fracture–indeed, several single-centre case series have reported increased bone mineral density after parathyroidectomy[34–36]; improvements in erythropoietin-resistant anaemia in patients with marked hyperparathyroidism [37–39]; and (3) improvements in nutritional status and humoral and cellular immunity [14, 40].

Other potential benefits beside “biochemical” improvement might be increased patients QoL, related to the improvement of pruritus, joint and bone pain, or muscle weakness. Although these effect were described in some case series, very regrettably none of the studies included in this meta-analysis specifically and reliably reported important changes in clinical symptoms. A recently published systematic review reported improved QoL in patients treated with sPTX for ESKD-related HPT, whereas cinacalcet did not [41]. However, the difference of impact between sPTx and cinacalcet on QoL has not been compared directly in head-to-head studies.

The immediate and obvious need now is for a well-conceived, well-conducted, independent RCT comparing sPTX with non-surgical therapy for SHPT associated with CKD, ideally over a 5 years time horizon, and featuring many different outcome strata, from mortality, major morbidity, to mental and physical health, QoL, and pharmaco-economics. To the best of our knowledge there is only one published RCT compairing cinacalcet with parathyroidectomy, which included 30 kidney transplanted patients with tertiary hyperparathyroidism and less severe CKD (eGFR>30 ml/min per 1.73 m^2÷^) [42]. At the end of the follow-up period (12 month), surgery induced greater reduction of iPTH and was associated with a significant increase in femoral neck bone mineral density; vascular calcification remained unchanged in both groups. Another randomized study comparing ultrasonic ablation for the treatment of SHPT with active vitamin D has been completed (ClinicalTrials.gov Identifier: NCT01640184), but these results are not yet published. Currently there is an ongoing RCT comparing cinacalcet with parathyroidectomy in peritoneal dialysis patients and is estimated to be completed by the end of 2017 (ClinicalTrials.gov Identifier: NCT01447368). These studies may help to reduce uncertainty in this area.

Despite an increasing PTH level in CKD patients over the last 15 years, recent data from DOPPS show a decreased rates of parathyroidectomy in all regions[7]. The rise in prescription rates for medications such as cinacalcet and vitamin D analogs, along with higher PTH targets and specifying indications for parathyroid surgery in recent nephrology guidelines [29], have most likely contributed to the decline in sPTX rates. Whether this is an overall benefit to patients remains unclear. Conversely, in Japan there was an increased trend for parathyroidectomy over time [43], probably due to the lower target range for intact PTH in Japanese guidelines than in guidelines used in other countries or to the fact that calcimimetics were available in Japan many years after their approval in Europe or in US.

This meta-analysis has several limitations. The most important of these is the observational design of the included studies with variable duration of follow-up, different indication for sPTX in different areas around the globe, and the variable matching criteria for the control group. The latter received “standard” medical therapy, consisting mostly of vitamin D compounds and/or phosphate binders [16–18, 20–22]; regrettably, some studies did not report any data regarding the treatment of the control group [23, 24, 44–48]. No study mentioned any data about calcimimetic treatment in the included patients; this though is most likely to be related to the fact that at the time of enrolment in these studies, cinacalcet was not yet available in many countries. This meta-analysis was also limited by the methodological quality of studies included; while there was some degree of heterogeneity between studies included in this meta-analysis, most of it could be explained by differences in the methodological quality of the trials. It was not possible to assess thermal, alcohol, or ultrasonographic ablation of parathyroid glands, or, the different surgical options (total vs. subtotal; autoimplantation) in this analysis. Renal transplantation was considered criteria of exclusion in all the included individual studies with one exception where sPTX was not associated with improved survival in patients with renal allograft [23]. This analysis lacked a detailed patient-level analysis of the clinical impacts of the surgery itself. There would most likely in real clinical conditions be some offset in overall benefit of the parathyroidectomy intervention as was showed in a recently analysis of the USRDS database where parathyroidectomy was associated with significant morbidity in the 30 days after hospital discharge and in the year after the procedure. However, due to the study design with the lack of a control group, the authors were not able directly to assess the impact on survival of sPTX [49].

Relatively the same survival benefit was also reported by Chen L. et al. in a recent metanalysis[50]. However, in contrast with this review, our metanalysis comprises 3 more studies with almost 5000 patients more, which may allow the decrease of confidence interval and give more strength to the overall analysis. Furthermore, we also did a analysis of the short-term mortality, and several subgroup analysis in order to explore heterogeneicity.

sPTX remains even in the modern nephrology era a valid and viable therapeutic intervention especially for long-term dialysis patients[7, 8]. Taken together, the results of this new meta-analysis suggests a beneficial effect of sPTX on all-cause and cardiovascular mortality, and maybe more importantly, challenges current practice that positions sPTX as last resort option when medical therapy fails. This meta-analysis (the largest to date) has attempted to gather all available and analyzable data on the impact of parathyroidectomy on hard endpoints–both short and long term all cause and cardiovascular mortality for renal patients with secondary hyperparathyroidism. The case for a properly conducted trial comparing parathyroid surgery with the combination of calcimimetics and vitamin D is now very strong, and the only way to settle this issue.

## Supporting information

S1 Table Search strategies. (DOCX)

S2 Table PRISMA checklist. (DOCX)

S1 Fig Cumulative metanalysis for all cause mortality. (EPS)

S2 Fig Cumulative metaanalysis for CV mortality. (EPS)
