# Supplementary material for: Impact of surgical parathyroidectomy on chronic kidney disease-mineral and bone disorder (CKD-MBD) – A systematic review and meta-analysis
Source: PLoS One. 2017 Nov 6;12(11):e0187025. doi: 10.1371/journal.pone.0187025 (PMC5673225; doi:10.1371/journal.pone.0187025)
Supplement: S1 Table — (DOCX) [file pone.0187025.s001.docx]

**S1 Table** Search Strategies:

| Database | Search terms used |
| --- | --- |
| Medline | 1. Parathyroidectomy/  2. parathyroidectomy.tw.  3. Parathyroid Glands/su [Surgery]  4. Parathyroid Glands/tr [Transplantation]  5. Surgical Procedures, Operative/  6. Parathyroid Glands/  7. 5 and 6  8. (parathyroid adj5 (surgery or surgical or transplant$ or autotransplant$)).tw.  9. or/1-4,7-8  10. Renal Osteodystrophy/  11. renalosteodystroph*.tw.  12. renal bone disease$.tw.  13. renal bone disorder*.tw.  14. renalSHPT.tw.  15. kidney bone disease*.tw.  16. or/10-15  17. Kidney Diseases/  18. exp Renal insufficiency, Chronic/  19. exp Renal Dialysis/  20. (kidney disease or kidney failure or renal disease or renal failure).tw.  21. (CKD or CKF or CRF or CRD or ESKD or ESKF or ESKD or ESRF).tw.  22. dialysis.tw.  23. (hemodialysis or haemodialysis or hemofiltration or haemofiltration or hemodiafiltration or  haemodiafiltration).tw.  24. or/17-23  25. 9 and (16 or 24) |
| EMBASE | chronic kidney failure'/exp OR 'chronic kidney disease' OR 'chronic kidney disorder' OR 'chronic kidney failure' OR 'chronic kidney insufficiency' OR 'chronic nephropathy' OR 'chronic renal disease' OR 'chronic renal failure' OR 'chronic renal insufficiency' OR 'kidney chronic failure' OR 'kidney disease, chronic' OR 'kidney failure, chronic' OR 'kidney function, chronic disease' OR 'renal insufficiency, chronic' AND ('parathyroidectomy'/exp OR 'parathyroidectomy') AND ('mortality'/exp OR 'excess mortality' OR 'mortality' OR 'mortality model') |
| CENTRAL | 1. parathyroidectomy:ti,ab,kw  2. MeSH descriptor Parathyroid Glands, this term only with qualifier: SU  3. MeSH descriptor Parathyroid Glands, this term only with qualifier: TR  4. (parathyroid NEAR/5 (surgery or surgical or transplant* or autotransplant*)):ti,ab,kw  5. parathyroid:kw and surgery:kw  6. (#1 OR #2 OR #3 OR #4 OR #5)  7. renal osteodystrophy:ti,ab,kw  8. renal next bone next disease*:ti,ab,kw  9. (renal next secondary next hyperparathyroid*):ti,ab,kw  10. kidney next disease*:ti,ab.kw  11. "kidney failure":ti,ab,kw  12. dialysis:ti,ab,kw  13. (hemodialysis or haemodialysis or hemofiltration or haemofiltration or hemodiafiltration or  haemodiafiltration):ti,ab,kw  14. (CKD or CKF or CRF or CRD or ESKD or ESKF or ESKD or ESRF):ti,ab,kw  15. (#7 OR #8 OR #9 OR #10 OR #11 OR #12 OR #13 OR #14)  16. (#6 AND #15) |
| www.clinicaltrials.gov | Parathyroidectomy |
